# The Use of Ultrasound for Detecting the Association Between Endothelial Dysfunction and lp13.3 Genomic Region rs646776 Polymorphism in Patients With Rheumatoid Arthritis From the Suez Canal Region

**DOI:** 10.7759/cureus.34743

**Published:** 2023-02-07

**Authors:** Afaf Ahmed, Aziza Omar, Maivel Ghattas, Mona Ghaly, Mohammad al-Shatouri

**Affiliations:** 1 Rheumatology, Suez Canal University Hospital, Ismailia, EGY; 2 Faculty of Medicine, Port Said University, Port Said, EGY; 3 Diagnostic and Interventional Radiology, Suez Canal University Hospital, Ismailia, EGY

**Keywords:** lp13.3 genomic region–rs646776 polymorphism, endothelial dysfunction, cardiovascular risk, rheumatoid arthritis, ultrasound

## Abstract

Background

Rheumatoid arthritis (RA) is an autoimmune disease associated with endothelial dysfunction (ED) and vascular morbidity. The study aimed to use ultrasound to assess the relationships of lp13.3 genomic region-rs646776 polymorphism with ED and subclinical cardiovascular disease (CVD) in patients with RA from the Suez Canal region in Egypt.

Results

This case-control study included 66 patients with RA and 66 healthy controls. Polymerase chain reaction-restriction fragment length polymorphism showed that the genotype frequencies for lp13.3 genomic region-rs646776 polymorphism in the RA group were 62.1% (n = 41), 34.8% (n = 23), and 3% (n = 2) for the AA, AG, and GG genotypes, respectively. The prevalence of the G allele was higher in the RA group than in the control group (20.5% and 7.6%, respectively; *p* < 0.01). Furthermore, ED was more prevalent in G allele carriers than in A allele carriers, suggesting a greater probability of ED and CVD in patients with RA with the GG genotype than in those with other genotypes.

Conclusions

This study indicated the validity of ultrasound in detecting the association between lp13.3 genomic region-rs646776 polymorphism and ED in Egyptian patients with RA. These findings could help identify high-risk patients with RA who may benefit from active treatment to help prevent CVD.

## Introduction

Rheumatoid arthritis (RA) is a chronic autoimmune disease that mainly affects peripheral joints, leading to systemic arthritis [1]. RA causes accelerated atherosclerosis and potentiates cardiovascular disease (CVD) [2]. The prevalence rates of smoking, hypertension, and dyslipidemia among Egyptian patients with RA are 7.1%, 25%, and 11%, respectively [3]. Ischemic heart disease is the most common cause of mortality in patients with RA [4]. Nontraditional risk factors, such as extra-articular manifestations, swollen joint count, autoantibody levels, and C-reactive protein (CRP) levels, have been implicated in accelerated atherosclerosis. Endothelial dysfunction (ED) is related to inflammation and can be assessed by flow-mediated dilatation (FMD) using high-resolution ultrasound of the brachial artery. This approach is a noninvasive and sensitive tool for evaluating subclinical atherosclerosis [5].

The results of a genome-wide association study identified several gene regions that significantly affect plasma cholesterol and CVD [6]. These genes are located on chromosome lp13.3 and play roles in ED and CVD complications. Moreover, polymorphism of the genomic region-rs646776 within the cadherin endothelial growth factor (EGF) LAG seven-pass G-type receptor 2 (CELSR2), proline/serine-rich coiled-coil 1 (PSRC1), and sortilin 1 (SORT1) gene cluster is related to dyslipidemia and atherosclerosis [7].

Therefore, in this study, evaluation of the genomic region-rs646776 polymorphisms within the CELSR2/PSRC1/SORT1 gene region and their association with ED detected by ultrasound was done in patients with RA. The current findings may provide insights into the identification of high-risk patients with RA who may benefit from active treatment to help prevent the occurrence of CVD.

## Materials and methods

Study design and patients

This study was carried out as a case-control study to determine the relationships of lp13.3 genomic region-rs646776 polymorphism with ED in patients with RA without clinically evident CVD through the use of ultrasound. The study compared patients with RA and healthy controls. This study was carried out at the Radiology Department, Suez Canal University Hospital, Ismailia, Egypt, over a five-year period. The study was approved by the Suez Canal University Ethical Research Committee & Institutional Review Board in accordance with the Declaration of the World Medical Association (Approval number: 887). Written informed consent was obtained from all of the participants in both study groups.

The population included in the study was classified into two groups: the study group (RA group without clinically evident CVD), which included 66 patients diagnosed with RA according to the American College of Rheumatology criteria, and the control group, which included 66 healthy volunteers who were matched to the study group according to their age and sex. Patients of both sexes, aged 18 years or more, who exhibited no symptoms and signs of cardiac disease were included in the study group. The following participants were excluded from the study: patients with comorbid conditions (e.g., diabetes mellitus and hypertension), obese individuals (BMI ≥ 30 kg/m^2^), pregnant individuals, and patients with a smoking history, patients with evidence of cardiac disease based on history, symptoms, clinical signs, investigations, or medications,.

Clinical and laboratory investigations

Full medical histories of the patients were assessed, and clinical examinations were performed. Outcome measures included the disease activity score (DAS) 28, which is a combined index used for global assessment of RA disease activity. The following parameters were included in the calculation: the number of swollen tender joints (0-28), erythrocyte sedimentation rate (ESR), and patient global health assessment (visual analog scale, 100 mm line, 0 = normal health and 100 = poor health). The levels of disease activity according to DAS28 were assessed as follows: inactive disease activity (remission), DAS28 < 2.6; low disease activity, DAS28 = 2.6-3.1; moderate disease activity, DAS28 = 3.2-5.1; severe disease activity, DAS28 ≥ 5.1.

Laboratory investigations included complete blood analyses, ESR, C-reactive protein (CRP), lipid profiles, rheumatoid factor, liver enzymes, and fasting and post-prandial blood sugar levels.

Imaging studies

**Figure 1:**
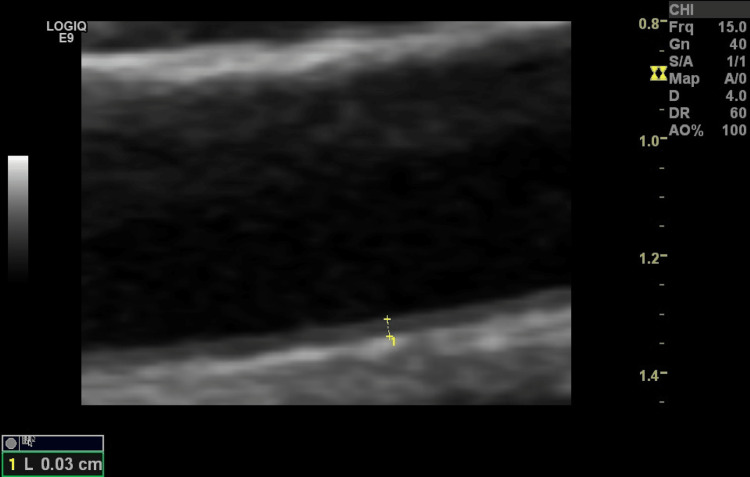
Longitudinal ultrasound image of an artery showing intima-media thickness.

**Figure 2:**
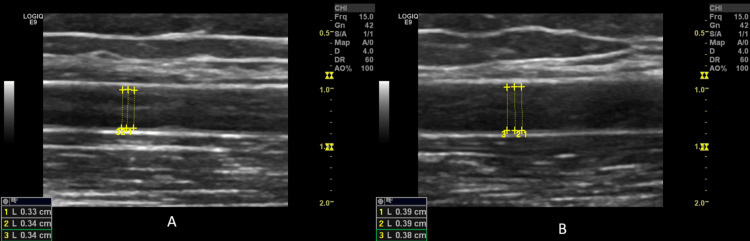
Longitudinal ultrasound images of the brachial artery showing placement of calipers for diameter measurement before (A), and 45-60 seconds after the deflation of the cuff (B). Endothelium-dependent flow-mediated dilatation; the endothelium-dependent vasodilation response was 5%.

**Figure 3:**
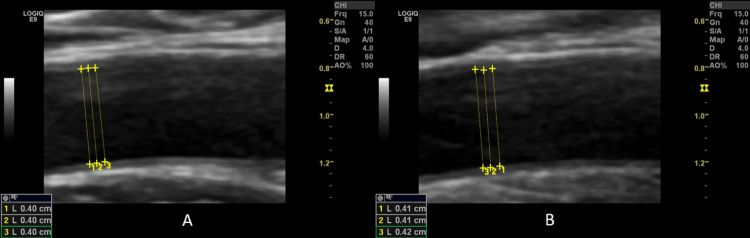
Longitudinal ultrasound images of the brachial artery showing placement of calipers for diameter measurement before (A), and 45-60 seconds after the deflation of the cuff (B). Impaired endothelium-dependent flow-mediated dilatation; the endothelium-dependent vasodilation response was 1.33%.

Imaging studies included chest X-ray for detection of any chest and/or heart abnormalities (pleural or pericardial effusion, pneumonia, pneumonitis, interstitial lung, nodules, or others) and Doppler ultrasound of the brachial artery to detect ED. Endothelium-dependent vasodilation was initiated by reactive hyperemia, whereas endothelium-independent vasodilation was initiated by sublingual administration of glyceryl trinitrate (GTN) tablets (Angised 0.5 mg tablet; GSK plc, Brentford, United Kingdom). The border between the intima and the blood was used. The distance between the outer intima and inner intima was checked three times for every measurement, and an average was obtained. The longitudinal section of the right brachial artery was used (Figure 1). The measurements were performed in a quiet room at a temperature of 21-22°C. Hitachi Preirus ultrasound machine (Hitachi, Ltd., Tokyo, Japan) and GE Logiq E9 ultrasound machine (GE HealthCare Technologies Inc., Chicago, Illinois, United States) having linear superficial probes with a frequency of 5-15 MHz were used. Patients were placed in a supine position for 15 minutes before measurements. An initial measurement was conducted (pre-FMD). Furthermore, a pneumatic cuff was inflated up to 200 mm Hg for four minutes. The arterial diameter was obtained again 45-60 seconds after the deflation of the cuff (post-FMD) (Figures 2, 3). After 15 minutes (to allow recovery before testing endothelium-independent relaxation), another diameter baseline measurement was conducted (pre-GTN). Next, a tablet of GTN was administered sublingually. The brachial artery diameter was measured three to four minutes later (post-GTN). A single radiologist with 18 years of experience (MA), who was blinded to the participants’ disease status, performed the ultrasound. FMD, GTN-mediated dilatation (GTNMD), and dilatation ratio were calculated as follows: FMD = ((post-FMD arterial diameter - pre-FMD arterial diameter)/pre-FMD arterial diameter) × 100; GTNMD = ((post-GTN - pre-GTN)/ pre-GTN) × 100 [8,9]. Each diameter was measured three times on the ultrasound image captured during the maximum diastolic phase, and the mean value was used to reduce the intra-observer variation. A cut-off value of < 7% for FMD was used to classify the response as low. DNA extraction, DNA quality assessment, amplification, and genotyping were done according to well-known laboratory methods [10,11].

Statistical analysis

All statistical analyses were carried out using IBM SPSS Statistics for Windows, Version 19.0 (Released 2010; IBM Corp., Armonk, New York, United States). Values are expressed as mean ± SD. Relationships between categorical variables were assessed using Pearson’s Chi-square test. Results with p-values less than 0.05 were considered statistically significant.

## Results

Table 1 shows the basic characteristics of the patients in the RA and control groups. The mean ages of individuals in the RA and control groups were similar (38.6 ± 7.3 and 36.6 ± 8.0 years, respectively; p > 0.05). The frequency of women was higher than that of men in both groups (women/men = 3:1), and there were no significant differences in sex between groups (p > 0.05). Moreover, no significant differences in occupation were observed between groups (p > 0.05). Patients with RA had a significantly higher prevalence of positive family history than the healthy control group (33.3% and 9.1%, respectively; p < 0.01).

**Table 1:** Demographic characteristics of the study groups. *significant p-value at < 0.05; **highly significant p-value at < 0.01 t: Student’s t-test; χ2P: Pearson Chi-square test; R: rheumatoid arthritis

Variables	RA group (n = 66)		Control group (n = 66)		Statistical test	p-value
Age (years)						
Mean ± SD	38.6 ± 7.3		36.6 ± 8.0		t = 1.6	0.11
Range	26–53		22–54			
Sex, n (%)						
Male	17 (25.8%)		16 (24.2%)		χ^2P ^= 0.04	0.84
Female	49 (74.2%)		50 (75.8%)			
Occupation, n (%)						
Unemployed	45 (68.2%)		43 (65.2%)		χ^2P ^= 0.31	0.58
Employed	21 (31.8%)		23 (34.86%)			
Family history, n (%)						
Negative	44 (66.7%)		60 (90.9%)		χ^2P ^= 11.6	0.0007**
Positive	22 (33.3%)		6 (9.1%)			

The disease duration in more than half of the patients (53.0%) was between five and 10 years. The mean values of ESR, CRP, and DAS28 in patients with RA were high (32.6 ± 9.7 mm/h, 15.3 ± 1.6 mg/L, and 4.5 ± 0.89, respectively). The majority of patients had elevated ESR and CRP levels (83.2% and 78.8%, respectively). Approximately 3% of the study patients showed disease remission. By contrast, 13.6%, 68.2%, and 18.2% of patients with RA showed low, moderate, and severe disease activities, respectively.

The most frequent symptom of RA was fatigue (54.5%), followed by dyspepsia (43.9%), subcutaneous nodules (36.4%), dryness of eyes (34.8%), and tingling and numbness (33.3%). Approximately 77%, 71.2%, 51.5%, and 47.0% of patients with RA were treated with hydroxychloroquine and nonsteroidal anti-inflammatory drugs, steroids, methotrexate, and leflunomide, respectively.

Table 2 shows the ultrasound assessment of FMD in the study groups. Significantly lower mean values of dilation after sublingual GTN (GTN %) and FMD% were observed in the RA group than in the control group (p < 0.01). The frequency of FMD < 7% was significantly higher in the RA group than in the control group (13.6% versus 3.0%, respectively; p < 0.05).

**Table 2:** Endothelial function using dilatation percentage in the study groups. *Significant p value at < 0.05; **Highly significant p value at < 0.01 t: Student’s t-test; χ2P: Pearson chi-square test; FMD: flow-mediated dilatation; GTNMD: glyceryl trinitrate-mediated dilatation; RA: rheumatoid arthritis

Variables	RA group (n = 66)		Control group (n = 66)		Statistical test	p-value
GTNMD %						
Mean ± SD	16.4 ± 4.2		18.8 ± 3.5		t = 3.6	0.001**
Range	8–26		10.5–26.5			
FMD %						
Mean ± SD	11.4 ± 3.2		12.7 ± 2.6		t = 2.8	0.007**
Range	5–19		6–16			
FMD < 7%	9 (13.6%)		2 (3.0%)		χ^2P ^= 4.9	0.027*

Table 3 shows the genotype and allele frequencies of lp13.3 genomic region-rs646776 in both study groups. The RA group had a significantly higher frequency of the AG genotype than the control group (34.8% and 15.2%, respectively; p < 0.01). Three percent of patients with RA had the GG genotype, whereas no patients in the control group had this genotype (p > 0.05). The RA group had a significantly higher frequency of the G allele than the control group (20.5% and 7.6%, respectively; p < 0.01).

**Table 3:** Genotype frequencies of lp13.3 genomic region–rs646776 in the study groups. *Significant p-value at < 0.05; **highly significant p-value at < 0.01 χ2P: Pearson Chi-square test; RA: rheumatoid arthritis

Variables	RA group (n = 66)		Control group (n = 66)		Used test	p-value
Genotypes, n (%)						
AA	41 (62.1%)		56 (84.8%)		χ^2P ^= 8.8	0.003**
AG	23 (34.8%)		10 (15.2%)		χ^2P ^= 6.8	0.009**
GG	2 (3.0%)		0 (0.0%)		Fisher	0.079
Alleles, n (%)						
A	105 (79.5%)		122 (92.4%)		χ^2P ^= 9.1	0.002**
G	27 (20.5%)		10 (7.6%)			

Table 4 shows the consistency of the observed genotype frequencies with Hardy-Weinberg equilibrium in the RA group. The differences in genotype frequencies between the observed values (RA group) and expected values (reference values) were not significant, indicating the consistency of the observed genotype frequencies of the RA group with Hardy-Weinberg equilibrium.

**Table 4:** Genotype distribution and Hardy-Weinberg equilibrium in the RA group (n = 66). The genotype frequencies of rs646776 polymorphism in the lp13.3 region were in accordance with the Hardy-Weinberg equilibrium. Comparisons were performed using χ2 tests. RA: rheumatoid arthritis

Genotypes	Observed values	Expected values	χ^2^	p-value
AA	41	41.8	0.33	0.56
AG	23	21.5		
GG	2	2.8		
Variant allele frequency	0.20			

Table 5 shows GTN% and FMD% among Genotype AA and Genotype AG/GG in RA patients. There is a statistically significant difference between the two groups. Low FMD (< 7%) was noted in 36% of Genotype AG/GG compared to 0% in Genotype AA. 

**Table 5:** Endothelial functions according to Ip13.3 genomic region-rs646776 in RA patients (n-66) ** Highly significant p-value at < 0.01 t: Student’s t-test; Fisher: Fisher’s exact test; GTN: glyceryl trinitrate; FMD: flow-mediated dilatation

Variables	Genotype AA (n=41)	Genotype AG/GG (n=25)	Used test	p-value
GTN%				
Mean ± SD	18.7 ± 3.2	12.5 ± 2.3	T=8.4	< 0.0001 **
Range	13.5-26	8-15.9		
FMD%				
Mean ± SD	13.2±2.3	8.5 ± 2.1	T=6.5	< 0.00001**
Range	9.7-19	4.5-12		
FMD < 7%	0 (0.0%)	9 (36.0%)	Fisher	0.00006**

## Discussion

This study included 66 patients with RA without clinically evident CVD and 66 healthy controls; both groups were subjected to ultrasound evaluation of ED to assess its relation to lp13.3 genomic region-rs646776 polymorphism. Both groups were matched for age and sex.

Several studies have investigated the associations of multiple genes with traditional vascular risk factors, CVD, and mortality in patients with RA [12-14]. The current results showed a significant reduction in the mean values of dilation detected by ultrasound of sublingual GTN (GTN%) and FMD% in the RA group compared with those in the control group. These results were in agreement with several previous studies, in which FMD and dilatation ratio were found to be significantly lower in patients with RA than in controls [15,16]. A similar Egyptian study conducted by Elshereef et al. reported significantly lower endothelial function parameters, including post-FMD, FMD% dilatation, and dilatation ratio, among patients with RA than among controls [16]. ED, indicated by impaired FMD, has been observed in the RA population, including in patients with no traditional CVD risk factors, at both the early and late stages of the disease. Moreover, ED appears to be related to RA severity [17-19].

The most replicated region for risk of CVD is located near lp13.3. Numerous studies have concluded that a variant polymorphism within or near the lp13.3 region is associated with a higher risk of coronary artery disease (CAD) and myocardial infarction as well as the overall risk of CVD and ED [20]. The association between ED or subclinical atherosclerosis and two gene polymorphisms (rs646776 and rs599839) in the lp13.3 genomic region was observed in several studies. Berry et al. examined the associations of these two single nucleotide polymorphisms (SNPs) (rs646776 and rs599839) with CAD and found that these SNPs were associated with an increased lifetime risk of CVD [21].

In patients with RA, López-Mejías et al. studied polymorphisms at lp13.3 genomic region-rs599839 [2]. They showed that patients with RA carrying the G allele exhibited greater ED (FMD%: 4.61%) than patients carrying the A allele (FMD%: 6.01%). Their results demonstrated a correlation between the rs599839 polymorphism and ED in RA. In the current study, significantly lower mean values of GTN% and FMD% were observed among patients with RA carrying the AG/GG genotypes than in those carrying the AA genotype. Moreover, 36% of patients with the AG/GG genotypes showed FMD < 7%, indicating significant ED. Ultrasound was the prime modality to detect ED and its association with genotype.

This study had several strengths. First, it was a case-control study, which is advantageous when studying dynamic populations, such as patients with RA and CVD. Furthermore, the study design was appropriate for evaluating diseases with a long latency period between etiological factors (e.g., generic susceptibility) and disease manifestation. Another strength of the study is the ethnic and racial homogeneity of the Egyptian population with similar genetic backgrounds and allele frequencies. Because the overall RA and CVD risk varies among different populations (i.e., Egyptian, American, European, and Asian), long-term longitudinal studies using large cohorts with multiple subgroups are required to ensure the generality of the results. The use of ultrasound for detecting ED was objective and reliable. However, one limitation of this study was the heterogeneity of manifestations of the study cohort, including various levels of biomarkers and different severities of symptoms and signs. The dependence on a single radiologist to perform the ultrasound procedure may also represent a bias.

## Conclusions

Ultrasound is a valid imaging modality to evaluate the relationships between lp13.3 genomic region-rs646776 polymorphism and ED. To the best of the authors' knowledge, this is the first Egyptian study that focused on the determination of the allele frequency of lp13.3 genomic region-rs646776 polymorphism in patients with RA without clinically evident CVD and healthy controls. Patients with RA carrying the G allele had significantly lower mean GTN% and FMD% values indicating the association between ED and G allele.

## References

[1] Feldmann M, Brennan FM, Maini RN (1996). Rheumatoid arthritis. Cell.

[2] López-Mejías R, González-Juanatey C, García-Bermúdez M (2012). The lp13.3 genomic region -rs599839- is associated with endothelial dysfunction in patients with rheumatoid arthritis. Arthritis Res Ther.

[3] El-Zorkany B, Mokbel A, Gamal SM, Mousa M, Youssef M, Hmamouchi I (2016). Comparison of comorbidities of the Egyptian rheumatoid arthritis patients to the global cohort of the COMORA study: a post-hoc analysis. Clin Rheumatol.

[4] Naz SM, Symmons DP (2007). Mortality in established rheumatoid arthritis. Best Pract Res Clin Rheumatol.

[5] Ter Avest E, Stalenhoef AF, de Graaf J (2007). What is the role of non-invasive measurements of atherosclerosis in individual cardiovascular risk prediction?. Clin Sci (Lond).

[6] Sandhu MS, Waterworth DM, Debenham SL (2008). LDL-cholesterol concentrations: a genome-wide association study. Lancet.

[7] Kjolby M, Andersen OM, Breiderhoff T (2010). Sort1, encoded by the cardiovascular risk locus 1p13.3, is a regulator of hepatic lipoprotein export. Cell Metab.

[8] Choi HY (2021). Carotid duplex ultrasound: interpretations and clinical applications. Ann Clin Neurophysiol.

[9] Ozdemir AO, Gulec S, Uslu N (2009). The relation between endothelial dependent flow mediated dilation of the brachial artery and coronary collateral development - a cross sectional study. Cardiovasc Ultrasound.

[10] López-Mejías R, García-Bermúdez M, González-Juanatey C (2011). Lack of association of IL6R rs2228145 and IL6ST/gp130 rs2228044 gene polymorphisms with cardiovascular disease in patients with rheumatoid arthritis. Tissue Antigens.

[11] Rodríguez-Rodríguez L, González-Juanatey C, Palomino-Morales R (2011). TNFA -308 (rs1800629) polymorphism is associated with a higher risk of cardiovascular disease in patients with rheumatoid arthritis. Atherosclerosis.

[12] Rodríguez-Rodríguez L, Lamas JR, Varadé J (2011). Plasma soluble IL-6 receptor concentration in rheumatoid arthritis: associations with the rs8192284 IL6R polymorphism and with disease activity. Rheumatol Int.

[13] Rodríguez-Rodríguez L, López-Mejías R, García-Bermúdez M, González-Juanatey C, González-Gay MA, Martín J (2012). Genetic markers of cardiovascular disease in rheumatoid arthritis. Mediators Inflamm.

[14] Sarwar N, Butterworth AS, Freitag DF (2012). Interleukin-6 receptor pathways in coronary heart disease: a collaborative meta-analysis of 82 studies. Lancet.

[15] Pereira IA, Laurindo IM, Zimmermann AF, Werner Castro GR, Mello F, Borba EF (2009). Single measurements of C-reactive protein and disease activity scores are not predictors of carotid atherosclerosis in rheumatoid arthritis patients. Acta Reumatol Port.

[16] Elshereef RR, Darwish A, Ali A, Abdel-Kadar M, Hamdy L (2015). Asymptomatic atherosclerosis in egyptian rheumatoid arthritis patients and its relation to disease activity. Int J Rheumatol.

[17] Kerekes G, Soltész P, Nurmohamed MT (2012). Validated methods for assessment of subclinical atherosclerosis in rheumatology. Nat Rev Rheumatol.

[18] Kerekes G, Szekanecz Z, Dér H (2008). Endothelial dysfunction and atherosclerosis in rheumatoid arthritis: a multiparametric analysis using imaging techniques and laboratory markers of inflammation and autoimmunity. J Rheumatol.

[19] Kerekes G, Soltész P, Dér H (2009). Effects of rituximab treatment on endothelial dysfunction, carotid atherosclerosis, and lipid profile in rheumatoid arthritis. Clin Rheumatol.

[20] Gonzalez-Gay MA, Llorca J, Palomino-Morales R, Gomez-Acebo I, Gonzalez-Juanatey C, Martin J (2009). Influence of nitric oxide synthase gene polymorphisms on the risk of cardiovascular events in rheumatoid arthritis. Clin Exp Rheumatol.

[21] Berry JD, Dyer A, Cai X (2012). Lifetime risks of cardiovascular disease. N Engl J Med.

